# Neurocritical Care in Brazil: Field of Practice or Subspeciality?

**DOI:** 10.1212/NE9.0000000000200256

**Published:** 2025-09-19

**Authors:** Thire Baggio Machado Marazzi

**Affiliations:** Department of Neurology, University of Sao Paulo Ribeirao Preto, Brazil.

Developing high-performance professionals in neurocritical care (NCC) is a unique challenge: cultivating advanced, multidisciplinary competencies across multiple medical specialties and applying them cohesively in real-time, bedside decision-making. Accordingly, training in this field requires the integration of essential knowledge from neurology, intensive care medicine (ICM), anesthesiology, and emergency medicine into a unified and practice-oriented framework.

From an epistemological standpoint, delineating when a consolidated body of knowledge constitutes a new scientific field, rather than an extension of existing disciplines, is inherently complex. This philosophical ambiguity also permeates the definitions of subspecialties, often determined by arbitrary regulatory criteria.

In the United States and Canada, NCC has been formally established as a subspecialty structured around shared core competencies^1^ and standardized board certification. Fellowship programs are accessible to physicians from diverse primary specialties, and although training pathways may be adapted to each fellow's background, they converge in forming specialists capable of providing comprehensive care for neurocritical ill patients.

But should this model be the standard for every country, particularly those whose primary specialties have far less exposure to NCC or limited numbers of trained professionals?

Consider the example of Brazil. Preliminary data^2^ indicate that fewer than 50% of neurologists and intensivists had any exposure to NCC during residency, and only 24% reported contact with neuromonitoring techniques, underscoring differences in primary training, critical care culture, and access to advanced technology compared with high-income countries. Beyond curricular limitations, the implementation of structured training programs must also contend with substantial regional heterogeneity, including disparities in infrastructure, workforce distribution, and access to specialized care.

Estimates indicate that fewer than 10 structured NCC training initiatives currently exist in Brazil. However, no formal curriculum has been defined by national medical societies, leading to significant variability in program length, content, and competencies**,** increasing the risk of producing specialists with substantial gaps in practical skills.

This lack of standardization poses a dilemma/strategic opportunity: Should Brazil prioritize consolidating NCC as a recognized subspecialty within neurology or ICM or seek recognition as a cross-disciplinary field of practice connecting multiple specialties on an equal footing?

Precedents exist. Palliative care and sleep medicine in Brazil faced similar barriers and ultimately achieved integration by becoming nationally recognized fields of practice. This approach enabled active collaboration among societies, facilitated the rapid expansion of training programs, and allowed for the inclusion of services within the public health system. It also simplified regulatory approval by the Federal Council of Medicine and enabled the national standardization of residency curricula and certification pathways.

This strategic alternative may also apply to other countries that, like Brazil, have a “blank slate” in NCC and persistent training gaps in primary specialties. A context-sensitive educational adaptation, often with lower costs and greater multiplier effects, could be implemented by fostering cross-specialty alliances, accelerating workforce growth, and raising the field's visibility. If adopted widely and supported by international forums that share experiences from diverse health care systems, this approach could help align global competencies while respecting local realities, ultimately strengthening both national capacity and the global identity of NCC.
